# A 3D micro-computed tomography study comparing embryonic skeletal development in layer versus broiler strains of the domestic chicken

**DOI:** 10.1016/j.psj.2024.104308

**Published:** 2024-09-06

**Authors:** Maeva Halgrain, Maris Schneider, Shumeng Jia, Agnès Narcy, Eric Gambier, Maxwell T. Hincke, Marc D. McKee, Sophie Réhault-Godbert, Natalie Reznikov

**Affiliations:** ⁎ INRAE, Université de Tours, BOA, 3780 Nouzilly, France; † Department of Anthropology, University of Western Ontario, London, Ontario N6A 3K7, Canada; ‡ Department of Bioengineering, McGill University, Montreal, Quebec H3A 0E9, Canada; § Departments of Innovation in Medical Education, and Cellular and Molecular Medicine, Faculty of Medicine, University of Ottawa, Ontario K1H 8M5, Canada; ║ Department of Anatomy and Cell Biology, McGill University, Montreal, Quebec H3A 0C7, Canada; ¶ Faculty of Dental Medicine and Oral Health Sciences, McGill University, Montreal, Quebec H3A1G1, Canada

**Keywords:** chorioallantoic membrane, eggshell, skeleton mineralization kinetic, tibiotarsus, mandible

## Abstract

Our objective was to analyze the effect of selection for divergent traits in the domestic chicken on embryonic skeletal development, which could affect postnatal bird welfare. Development was compared between the Ross 308 broiler line (fast growth and muscle mass accrual) and Novoponte layers (high laying rate and egg quality).

In Study 1 (Initial Conditions), we characterized egg composition prior to incubation and identified the onset of embryonic skeletal mineralization in the 2 strains. In Study 2 (Developmental Dynamics), we used 3D X-ray tomographic imaging (µCT) on incubation days ED11, ED13, ED15 and ED17 to track skeletal maturation trajectories as a pseudo-time series.

Results showed that Ross 308 embryos, which are heavier than Novoponte embryos, possess higher levels of yolk nutrients including phosphorus and calcium, but lower eggshell mineral content, than Novoponte embryos. Skeletal mineralization started synchronously in both strains, on ED11. The higher mineral ion content in the larger yolk of Ross 308 eggs compared to Novoponte eggs may partly explain why skeletal mineralization in Ross 308 embryos advances faster: using µCT, we show that the mandible and tibiotarsi in Ross 308 embryos are larger at ED11 and ED13 compared with Novoponte embryos. However, Novoponte embryos catch up from this initial lag in mineralization by ED15. The timing of the Novoponte acceleration coincides with the functional activation of the chorioallantoic membrane in releasing calcium from the inner eggshell. This correlates with a decrease in eggshell thickness from ED11 to ED17 in Novoponte eggs, which was not observed during Ross 308 incubation.

To conclude, while some temporal discrepancies exist in early skeletal development between the embryos of Ross 308 and Novoponte strains, overall prenatal skeletal maturation seems to be robustly regulated. Despite selection for antagonist production traits, layer and broiler prehatch skeletal morphology ultimately synchronizes.

Practically, since the extent of skeletal maturity equalizes between strains towards the end of incubation, refinements of farming practices, postnatal environment, and diet should be considered for improving domestic fowl welfare.

## INTRODUCTION

Egg-laying animals—including birds—have perfected extracorporeal embryonic development over hundreds of millions of years of evolution. An avian egg is an ingenious structure where the yolk contains an optimized package of nutrients, the egg white functions as a physicochemical barrier against pathogens and as a water reservoir, and the mineralized shell serves for mechanical protection, prevents desiccation, supplies calcium, and sometimes acts as a substrate for pigmentation and maculation patterns that provide a camouflage function (Birkhead, 2016).

The assembly of the different components of the egg follows a precise temporal and spatial sequence. The ovum/yolk forms in the ovary before the egg is fertilized and before it enters the oviduct (Romanoff and Romanoff, 1949). It takes several days for a female to produce the yolk (Astheimer and Grau, 1990), *e.g.,* about 14 day in the chicken (Daddi, 1896). The relative size of the yolk with respect to the entire egg varies amongst species (Sotherland and Rahn, 1987), where precocial species generally assemble relatively large yolks. Moreover, the yolks of replacement eggs in the guillemot take a shorter time to assemble than the yolk of the first-laid egg (Birkhead and del Nevo, 1987). In the duck, the composition of the yolk may reflect the female's perception of her mating partner, where a richer yolk is produced in response to a more attractive male (Cunningham and Russell, 2000). Albumen and eggshell membrane assembly takes place in the magnum and the white isthmus, respectively, as the egg travels down specialized regions of the oviduct (Romanoff and Romanoff, 1949). Finally, the mineralized eggshell is deposited on top of an organic fibrous membrane and with connections established through an intricate mineral attachment mechanism (Buss, Reznikov et al., 2023) in about 20 h in the shell gland (uterus) in the chicken. At laying (oviposition), the egg with its eggshell provides an enclosure sturdy enough to withstand the weight of its brooding parent. During the literally “precipitous” biomineralization process in the shell gland, the calcium required for the shell is supplied by osteoclast-mediated resorption of the hen's medullary bone (Bloom, Domm et al., 1958), which in turn is rapidly re-formed through mineral ion uptake procured from the diet. During incubation of an egg, dissolution of the innermost layer of the mineralized eggshell achieved through lowering of the local pH (as mediated by the cells of the chorioallantoic membrane), which is a major source of calcium for the growing chick skeleton (Chien, Hincke et al., 2008). The calcium-releasing shell dissolution process orchestrated by the chorioallantoic membrane (Halgrain, Georgeault et al., 2022) is functional by day 13 of domestic chicken incubation at the point where calcium supply from the yolk dwindles. By the time of hatching on day 21, the chick embryo is strong enough to break through the eggshell, and the eggshell thinning weakens it sufficiently enough to be broken from the inside. Thus, the embryo has all the components necessary for successful development, and its growth is robustly regulated both spatially and temporally (Romanoff, 1960).

Selective breeding of the domestic chicken enables to establish and propagate phenotypic traits, such as the quality and yield of table eggs or meat, in a way that is valuable for human nutrition. While layer chicken breeds with enhanced egg production have been known since the Middle Ages in Europe (*e.g*., the Leghorn strain) or even before current era in Cental Asia (Peters, Richter et al., 2024), broiler meat breeds became well-established during the middle of the 20th century in response to growing demands from the food industry. It is increasingly concerning today that the highly selected features of fast-growing broiler breeds puts domestic fowl out of synchrony with their finely tuned developmental timetable, thus contributing to high morbidity and mortality in the poultry industry. Indeed, accelerated body mass accrual in the broiler, coupled with a slower post-hatching pace of skeletal maturation, results in numerous musculoskeletal ailments, undermining the well-being of birds. While the hatchlings of layer and broiler chicken strains are similar in size (Williams, Solomon et al., 2000), it has been shown that during posthatch development the typical growth curve of the meat chicken dramatically bypasses that of the layer birds, resulting in a 3-fold higher comparative weight gain by 6 wk of age (Williams, Solomon et al., 2000). As a result of intensive selection for production phenotypes, both broilers and layers show musculoskeletal disorders. Layer hens with high-laying performance and concomitant export of calcium mineral often suffer from osteoporosis-related fractures (Benavides-Reyes et al., 2021a), while broilers have locomotion problems and leg weakness (Kestin, Knowles et al., 1992, Julian, 2005). Moreover, there is increasing evidence that metabolic differences between broiler and layer strains are detectable before hatching (Buzala, Janicki et al., 2015; Halgrain, Bernardet et al., 2023; Jia, Fulton et al., 2023; Bernardi, Ramé et al., 2024; Contriciani, Grade et al., 2024).

Given this context and the known differences in domestic chicken lines established by selective breeding, in the present study we provide a comparative *in ovo* analysis designed to follow the pre-hatch kinetics of skeletal growth in layer *versus* broiler chicken embryos. Our first study compares the initial conditions in both strains by analyzing the mineral ion and macronutrient content of the eggs, as well as the general growth of embryos around the onset of embryonic skeleton mineralization. In the second study we describe the dynamics of skeletal development *in ovo*. For this, we conducted a pseudo-time-lapse 3D imaging experiment between embryonic day (**ED**) 11 and ED17, given that skeletal mineralization in the chick embryo begins on ED11 and can be first visualized *in ovo* by X-ray microcomputed tomography (µCT) (Romanoff, 1960). Embryos of both lines at ED17 show skeletal development corresponding to developmental stage 35 (Hamburger and Hamilton, 1951; Freeman and Vince, 1974), where the yolk sac is still fully external to the body, the embryo is aligned with the long axis of the egg, and the beak is pointing towards the air sac.

## MATERIALS AND METHODS

### Ethics Statement

The experimental procedures complied with the European legislation on the “Protection of Animals Used for Experimental and Other Scientific Purposes” (2010/63/UE), and followed the guidelines approved by the Institutional Animal Care and Use Committee (**IACUC**). Research experiments complied with ARRIVE guidelines (Percie du Sert, Hurst et al., 2020).

### Study 1—Initial Conditions: Characterization of Eggs and Embryos from the Novoponte Layer and Ross 308 Broiler breeds

*Egg management.* Sixty fertilized eggs laid by 45-week-old broiler hens (Ross 308, Aviagen) were purchased from hatchery Boyé Accouvage (La Boissière en Gâtines, France), and sixty fertilized eggs from 33-week-old layer hens were purchased from a Novoponte hatchery (Novogen, La Chapelle-sur-Loire, France). Hens’ ages were selected based on the onset of stable egg production and availability (Izat, Gardner et al., 1985, Benavides-Reyes et al., 2021b). Eggs were stored for 5 to 6 d at 75% relative humidity and 16°C. Eggs were weighed, and batches of ten eggs of comparable weights were defined to avoid any bias related to the weight parameter. Eggs were all incubated in the same incubator at 37.8°C and 55% relative humidity for 9, 10, 11, or 12 days under standard conditions with automatic egg turning every hour using a Bekoto B64-S incubator (Pont-Saint-Martin, France).

*Egg and embryo characterization*. At incubation days 9, 10, 11, and 12 (ED9, ED10, ED11, and ED12, respectively), batches of ten eggs were removed from the incubator. Eggs were weighed and opened to collect and euthanize the embryo by cervical dislocation according to European legislation. Washed eggshells were dried in an oven for 2 h at 110°C to ensure the loss of bound water, and weighed. After weighing, embryos were processed to examine the mineralization of the skeleton by whole-mount staining with Alcian Blue and Alizarin Red S, as previously described (Ovchinnikov, 2009) and adapted (Halgrain, Bernardet et al., 2022). Briefly, before sampling/dissection, embryos were placed in hot tap water (65–70°C) for 30 sec. Skin, viscera, liver, kidney, and gut were removed. The embryos were then fixed in 95% ethanol for 4 d, transferred to acetone solution for 1 d and briefly rinsed in a bath of demineralized water. After this step, embryos were placed in an Alcian Blue solution (0.1 g/L; Sigma, Saint-Louis, ref. A5268) for 1 d followed by rehydration using baths of ethanol (3 baths; one 24 h after staining, and then 2 baths at 2-h intervals) for staining cartilage. After an overnight rinse in demineralized water, the embryos were placed in a 1% KOH solution for maximum 24 h (3 h for the youngest embryos and up to 24 h for the oldest ones). Counterstaining with Alizarin Red S solution at a concentration of 0.01 g/L (Sigma, Saint-Louis, ref. A5533) was performed for 5 h (ED9, 10, and 11) or about 18 h for embryos at ED12 to visualize mineralization sites in the skeleton. Stained embryos were rinsed in 20% glycerol /1% KOH solution before storage at 4°C in 50% glycerol/50%ethanol solution.

### Study 2—Developmental Dynamics: Investigation of Mineralization Trajectories in the Novoponte Layer and Ross 308 Broiler strains

*Egg management*. Sixty fertilized eggs laid by 33-week-old broiler hens (Ross 308) were purchased from Boyé Accouvage (La Boissière en Gâtines, France), and 60 fertilized eggs from 26-week-old Novoponte layer hens (Novobrown eggs) were purchased from a Novoponte hatchery (La Chapelle-sur-Loire, France). Hens’ ages were selected based on the onset of stable egg production and availability (Izat, Gardner et al., 1985, Benavides-Reyes et al., 2021). Eggs were maintained in the Poultry Experimental Facility (PEAT) UE1295 (INRAE, F-37380 Nouzilly, France, DOI: 10.15454/1.5572326250887292E12). Eggs were stored for 3 d at 75% relative humidity, at 16°C. Prior to incubation, 10 eggs per strain were collected to analyze various quality parameters, and to quantify calcium and phosphorus content in eggshell and yolk. Fifty eggs per strain were incubated for 11, 13, 15, or 17 d (ED11, ED13, ED15, and ED17, respectively) under standard conditions (55% relative humidity, at 37.8°C, with automatic egg turning every hour using a Bekoto B64-S apparatus (Pont-Saint-Martin, France). At each time point, 10 eggs were collected, refrigerated for 2 h at 4°C, followed by 24 h at −20°C, and then finally kept at −80°C until their use for microcomputed 3D imaging (see below).

*Measurement of egg quality parameters.* Egg weight prior to, and after, incubation was measured to evaluate the homogeneity of egg batches. Batches of eggs from the Novoponte strain (n = 10) and from the Ross 308 strain (n = 10) were analyzed for measurement of eggshell strength and equatorial thickness using the micrometer provided together with the digital egg tester DET6000 (Nabel, Japan), and for egg yolk and egg white weights. Eggshell weight was determined after rinsing eggshells with tap water and drying for 2 h at 110°C, similar to Study 1.

*Measurement of calcium and phosphorus content in eggshell and yolk*. Quantitative analysis of mineral ion content was performed as previously described (Halgrain, Bernardet et al., 2022). Yolks were collected by aspiration with a syringe, lyophilized and kept at −80°C. Eggshells were washed, dried, weighed and ground using a Cryomill ball mill (Retsch, Haan, Germany). Samples of yolk and eggshell powder (300 mg) were dissolved in 10 mL of 65% nitric acid and heated/digested in a microwave for 15 min at 200°C, 1800 W (Ethos Up, Milestone, Sorisole, Italy). Total phosphorus (**P**) and calcium (**Ca**) content was determined using an inductive coupled plasma atomic emission spectrometer (ICP OES ThermoscientificTM iCAPTM 7200; method 990.08; AOAC International, 2006). Standard solutions (P and Ca) were prepared from a 1,000 mg/mL stock solution (Certipur Merck, Darmstadt, Allemagne). All assays were performed in duplicates.

*X-ray microcomputed tomography (µCT).* For each embryonic day studied (ED11, ED13, ED15, and ED17), 32 eggs (59.6 ± 2.5 g) containing viable embryos were selected to include 4 eggs each for the different strain/embryonic day (**ED**) combinations. Embryos were euthanized by freezing intact eggs that were kept frozen until analysis. After thawing overnight, eggs were coated with a thin layer of transparent cyanoacrylate glue brushed onto the eggshell surface to stabilize them and prevent cracking and leakage of contents. Each entire/intact egg was placed with the blunt end oriented upwards in a 100 mL polyethylene measuring cup and stabilized with surrounding polystyrene foam wedges for subsequent *in ovo* analysis by µCT (imaging was performed using an Xradia Versa 520 µCT scanner; Carl Zeiss, Oberkochen, Germany). To capture the entire egg in a single field of view, a flat panel detector was used, with an 80 kV source voltage. Detector and source distances were optimized to create a voxel size between 50 and 55 µm (to accommodate the full egg) with ×2 binning (to optimize signal-to-noise ratio). At 2 seconds exposure per projection, 3001 projections were obtained in each acquisition, and the reconstructed 3D images were exported in the 3D TIFF format, 16-bit. One egg from each strain were lost to cracking and leaking, and 1 Ross 308 egg contained a runt embryo that was not included in the analysis. Accordingly, twenty nine 3D images were available for analysis, giving 3 to 4 eggs per strain/ED combination.

*3D image processing and analysis.* All digital image operations were conducted using Dragonfly software v2022.2 (Comet Technologies Canada Inc., Montreal, Canada—formerly Object Research Systems Inc.). Image segmentation was done using a standard 5-level U-Net, with 3 output classes: eggshell, skeleton, and background. In the XY (transverse) plane of each 3D image, every 100th 2D image was selected and exported as an abridged training input. The training input, comprising 1% of the full 3D image data, was manually segmented using combinations of local and global greyscale thresholding, and edge detection. Two operators (MS and SJ authors) monitored the accuracy of manual segmentation, which was then paired with the training input as the training output. Such multiple pairings of input and output comprised the training set, also called “ground truth”, for the U-Net training. The training sets were partitioned into learning (80%) and validation (20%) sets. Training patch size was 64 pixels, batch size was 64, and training continued until no loss function improvement was observed for 10 consecutive epochs. After initial segmentation, corrections were made on different slices, and the U-Net performance was further refined in an additional round of training following the same procedure. This deep-learning approach allowed for unsupervised segmentation of all 3D images with satisfactory accuracy (Reznikov, Buss et al., 2020).

Following automated segmentation of all 29 volumetric images, the eggshell and skeleton regions of interest (**ROI**) were analyzed separately, as binary images. The eggshell thickness was analyzed using the “volume thickness map” operation of the Dragonfly software. This operation computes inscribed imaginary spheres within 3D foreground features, and the spheres’ maximum diameters are mapped and color-coded in 3D. For an eggshell of approximately 7,000 mm^3^ surface area and 0.4 mm thickness, about 50,000 individual measurements were carried out automatically and exported as a histogram for statistical analysis in MATLAB, using a peak-fitting operation. Embryonic skeleton automated segmentations inevitably included small X-ray opaque mineral formations apparently associated with the yolk sac. These nonskeletal entities were mostly removed by morphological sorting, as part of the multi-ROI analysis plugin in Dragonfly. Multi-ROI analysis also converts multiple entities belonging to a single class (*e.g*., all “bones”—semantic segmentation) into individually color-coded identifiable objects (*e.g*., femur, parietal, mineral speckles—instance segmentation). Mandibles and right tibiotarsi were digitally isolated, and their maximal and minimal linear dimensions (Feret diameters) were calculated and exported. Growth progression was analyzed using the right tibiotarsus as a convenient early detectable element (Hamburger and Hamilton, 1951).

*Statistical analyses*. For egg and embryo characteristics, statistical analyses were performed using XLSTAT software (Data Analysis and Statistical Solution for Microsoft Excel, Addinsoft, Paris, France 2017). Following a normality test (Shapiro–Wilk test), statistical analyses were achieved using a Kruskal–Wallis test (P < 0.05) followed by a pair comparison using a Mann–Whitney test (*P* < 0.05), or a t-test (*P* < 0.05). For 3D image quantitative read-outs, a built-in t-test (*P* < 0.05) function under Data Analysis in Microsoft Excel was utilized. The 3D eggshell thickness measurements in CSV format were analyzed using PyCharm software v2019.3.3 (an integrated development environment by JetBrains s.r.o.) using Python 3.8. The histogram data was imported for programming as a DataFrame object through Pandas (version 1.3.2) and fitted by a self-defined Gaussian function implemented with NumPy (version 1.21.2). Data visualization was then carried out using Matplotlib (version 3.4.3).

## RESULTS

### Initial Conditions

**Figure 1:**
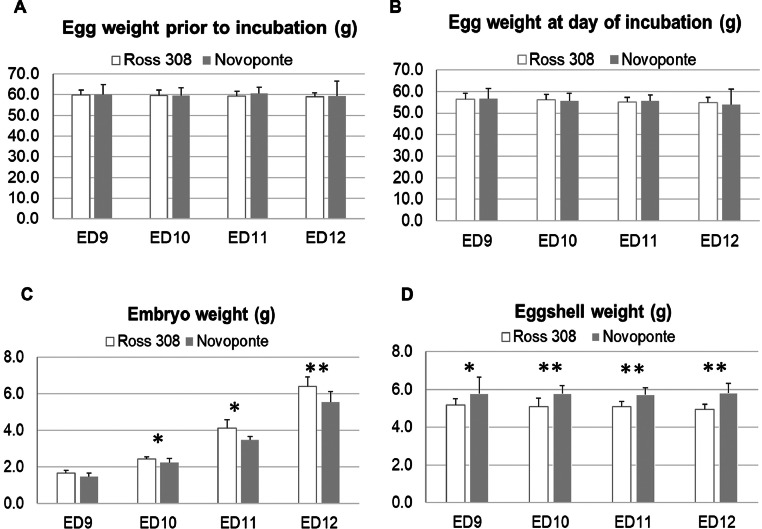
Egg and embryo characteristics of Ross 308 and Novoponte strains at 4 consecutive timepoints (ED9, ED10, ED11, and ED12). (A) Egg weight prior to incubation. (B) Egg weight after incubation for 9, 10, 11 and 12 d. (C) Embryo weight after incubation for 9, 10, 11, and 12 d. (D) Eggshell weight after incubation for 9, 10, 11 and 12 d. *P < 0.05; **P < 0.005.

*Comparison of Ross 308 and Novoponte eggs and embryos.* Batches of eggs (10 eggs per strain per developmental stage) were selected in a way such that egg weights were not statistically different (Figure 1A). No statistical difference was observed between egg batches after incubation at ED9, ED10, ED11 and ED12 (Figure 1B). However, Ross 308 embryos were heavier than Novoponte embryos from day E10 onwards (Figure 1C), while eggshell weights of Ross 308 eggs were lower than that of Novoponte eggs at all incubation times (Figure 1D).

**Figure 2:**
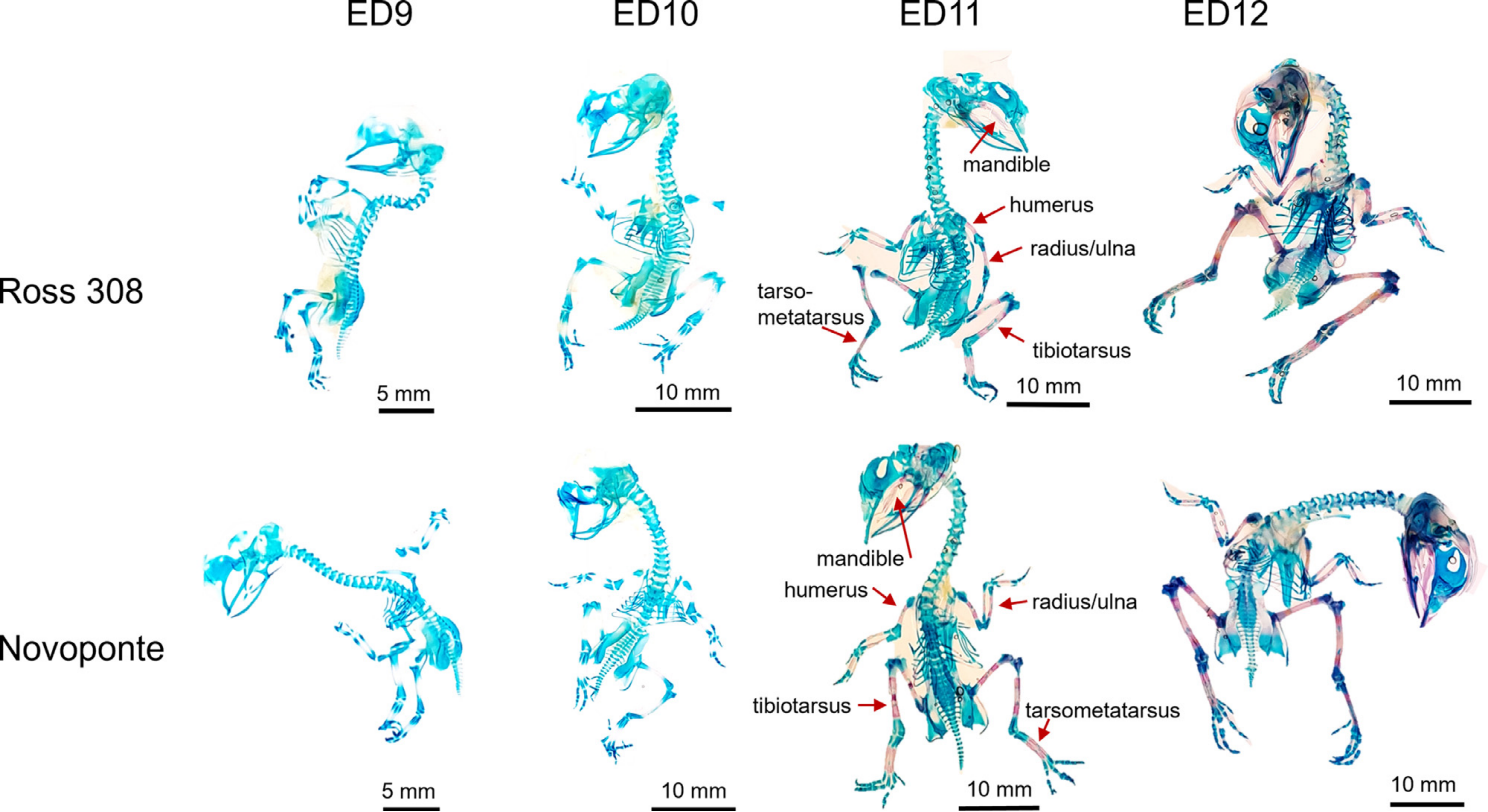
Staining of embryo skeletons with Alizarin Red S for calcium and Alcian Blue for proteoglycans at embryonic days ED9 to ED12. Mineralized tissues are stained in red while cartilage appears in blue. Red arrows at ED11 indicate the first ossification centres.

*Determination of the onset of skeletal mineralization in Ross 308 and Novoponte embryos.* Staining of embryos with Alcian Blue and Alizarin Red S did not reveal any overt visual differences in the timing of skeletal mineralization onset (Figure 2), in agreement with prior studies (Romanoff, 1960). Both Ross 308 and Novoponte embryos presented with Alizarin Red S staining on ED11 and ED12 while no red/pink staining was observed after 9 or 10 d of incubation in the midshafts of long bones and in the body of the mandible (Figure 2). Following this observation, the developmental stages of interest were identified for Study 2—Developmental Dynamics*.*

*Egg composition, and calcium and phosphorus content in Ross 308 and Novoponte strains, prior to incubation.* Batches of eggs (10 eggs per strain) were prepared so that egg weights were comparable (Figure 1A; *P* = 0.762, Table 1). While whole egg weight was not statistically different between strains, the Ross 308 eggs demonstrated a higher yolk weight, and, correspondingly, a higher yolk proportion. Moreover, Ross 308 eggshells demonstrated lower strength and thickness than Novoponte eggshells (Table 1). The content of phosphorus and calcium per gram of eggshell was not statistically different between the lines, whereas the mineral content per gram of yolk was higher in the Ross 308 eggs compared with Novoponte eggs. Ross 308 eggs possessed a higher proportion of yolk-to-egg white (*P* = 0.02, Table 1), and higher phosphorus and calcium per gram of yolk (*P* = 0.028). The higher weight of yolks combined with the higher calcium and phosphorus content in Ross 308 eggs contribute to the richer calcium and phosphorus reserves in the yolk in comparison to Novoponte eggs.

**Table 1:** Characteristics of Novoponte and ROSS 308 eggs prior to incubation (ED0).

Parameter	Novoponte	Ross 308	*P*-value
Egg weight ED0 (g)	60.25 ± 2.97	60.36 ± 4.04	0.762
Yolk weight (g)	14.71 ± 2.59	17.55 ± 1.31	**0.002**
Egg white weight (g)	45.54 ± 1.64	42.81 ± 3.04	**0.024**
Yolk %	12.33 ± 1.40	14.71 ± 0.66	**0.002**
Eggshell strength (N)	44.10 ± 6.70	36.12 ± 3.82	**0.004**
Eggshell weight (g)	5s.96 ± 0.55	5.22 ± 0.41	**0.003**
Eggshell thickness (mm)	0.42 ± 0.04	0.38 ± 0.02	**0.007**
P (mg/g dried eggshell)	1.10 ± 0.17	1.09 ± 0.12	0.846
Ca (mg/g dried eggshell)	368.82 ± 7.45	370.41 ± 8.27	0.674
P (mg/g lyophilized yolk)*	11.49 ± 0.42	11.92 ± 0.33	**0.028**
Ca (mg/g lyophilized yolk)*	2.97 ± 0.21	3.28 ± 0.32	**0.028**

⁎ The average weights of lyophilized yolk were 7.46 ± 1.23 g and 8.96 ± 0.67 g for Novoponte and Ross 308 eggs, respectively. Bolded font indicates statistical significance.

The higher eggshell weight in Novoponte eggs compared with Ross 308 eggs (5.96 ± 0.5 vs. 5.22 ± 0.4, *P* = 0.003) results in a higher amount of calcium (2168.21 ± 190.32 vs. 1918.83 ± 162.17 mg per eggshell, *P* = 0.009), but not of phosphorus (although there is a trend, 6.47 ± 1.13 vs. 5.61 ± 0.63 mg per eggshell, *P* = 0.064).

### Developmental Dynamics

**Figure 3:**
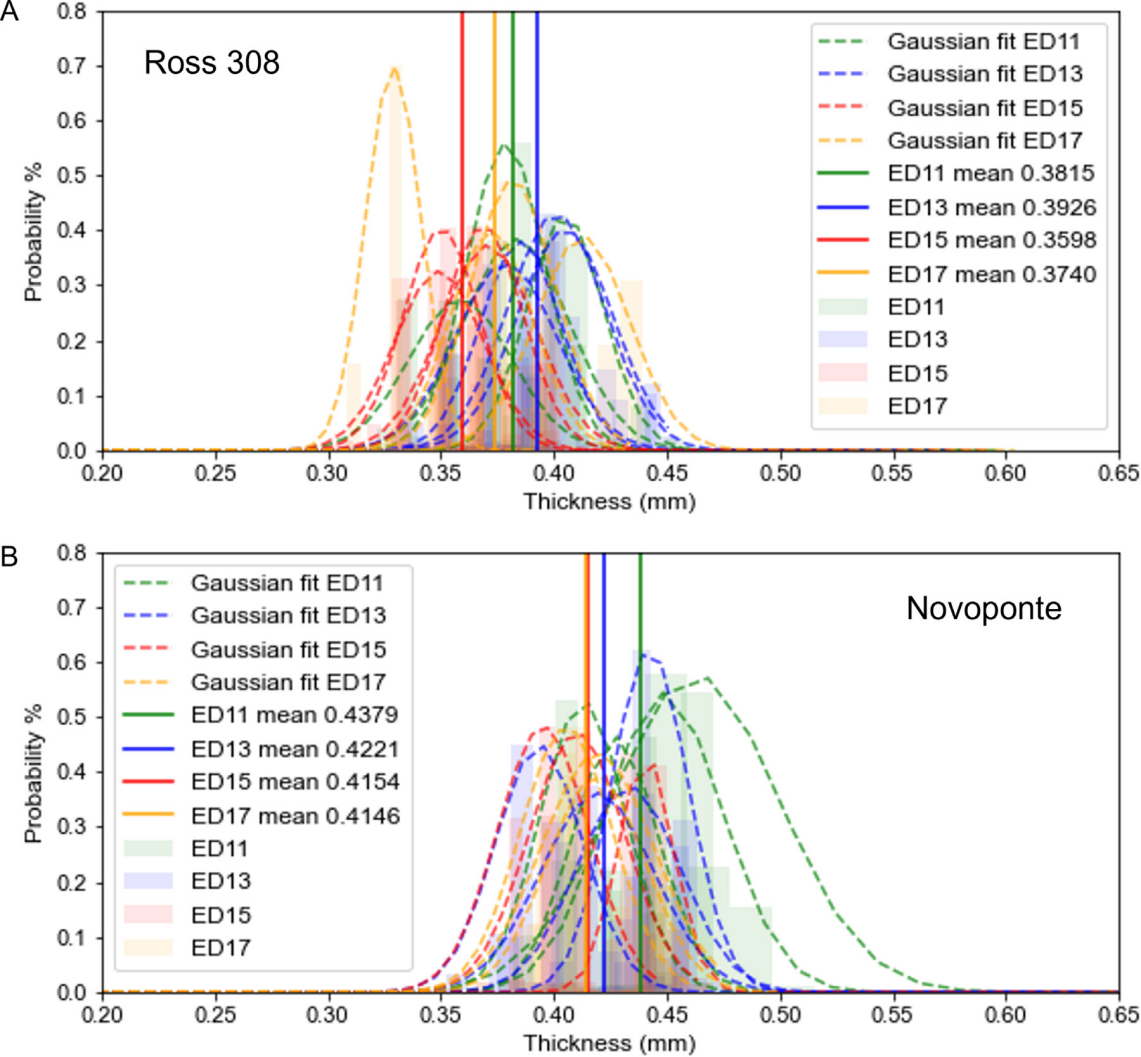
Digital measurements of the eggshell thickness in the Ross 308 (A) and Novoponte (B) eggs, obtained from 3D image analysis (segmentation and thickness mapping of the eggshell, µCT). All 29 eggs are presented in green for ED11, blue for ED13, red for ED15 and yellow for ED17 in both strains. Solid vertical lines indicate the mean eggshell thickness values for the 3 or 4 eggs for each developmental stage (ED), and dashed lines show the fitted Gaussian distribution of local digital eggshell thickness measurements. Pale background histogram bars indicate the abundance of local thickness measurements per 1 thickness map (*i.e*., per eggshell, approximately 50,000 measurements), and the substantial individual variation observed.

*Eggshell thickness during incubation.* Eggshell thickness histogram values for both strains on days ED11, 13, 15, and 17 are presented in Figure 3. Here, all 3D thickness measurements per eggshell were exported as histograms, Gaussian curves were fitted to the histograms, and group means were calculated. Two trends were observed. First, the Ross 308 eggshells are generally thinner throughout the incubation period. Second, the Novoponte eggshells demonstrate an orderly trend of the eggshell becoming thinner from day ED11 to day ED17, consistent with the notion of gradual dissolution of the eggshell mineral during incubation. In contrast, the Ross 308 eggshells did not demonstrate such a trend, likely because of their high individual and group variation since all avian eggs thin to some degree as part of egg incubation and hatching. In either line, no difference was observed in the eggshell thickness between the equatorial region and the rest of the eggshell at the X-ray microscopy resolution used in this study (voxel size about 50 µm).

**Figure 4:**
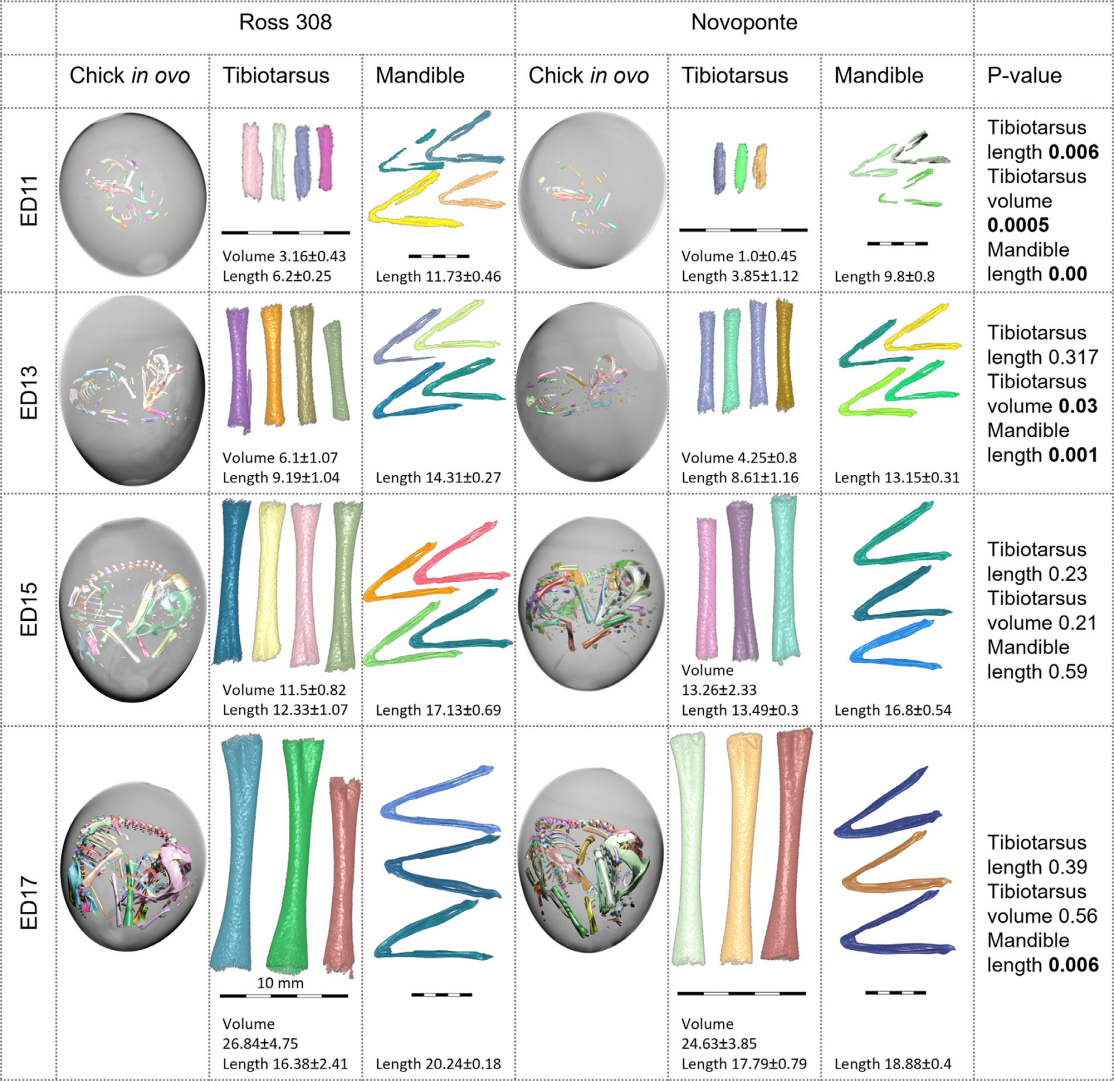
Volumetric rendering of the embryo *in ovo* (1 representative individual per group), of the tibiotarsi and the mandibles. For the mineralized portions of the mandibles and tibiotarsi, volume is reported in mm^3^, and length is in mm. Scale bars pertain to the respective columns. Difference between the 2 strains is statistically significant for tibiotarsi (volume and length) on days ED11 and 13, and for mandibles on days ED11, 13, and 17, but not on d 15. n = 4 eggs for Novoponte ED13 and Ross 308 ED11, ED13, ED15; n = 3 eggs for other groups, as shown.

*Tibiotarsus and mandible development during incubation.*Figure 4 presents a gallery of typical embryos *in ovo* and of all segmented skeletal elements in both strains. Only the mineralized portions of the mandibles and tibiotarsi can be visualized by µCT, whereas the distal and proximal ends of the tibiotarsus, and the proximal parts of the mandible remain unmineralized and therefore X-ray transparent (Romanoff, 1960). All tibiotarsus bones and all mandibles are scaled proportionately to the same scale bar per column. Note the rotation and re-orientation of the embryo along the egg axis at ED15 in both strains. Volumetric renderings of the entire embryos are presented in Supplemental Videos SV 1, and of the tibiotarsi and the mandibles in Supplemental Videos SV 2.

## DISCUSSION

### Developmental Trajectory

Our results show that the broiler strain Ross 308 and the layer strain Novoponte follow somewhat different prenatal developmental trajectories, which are summarized in Table 2 below.

**Table 2:** Summary of prenatal parameters of Ross 308 and Novoponte eggs and embryos (bold font indicates divergent observations).

	Parameter	Ross 308	Novoponte
Initial conditions	Egg weight	No difference observed	
	Eggshell weight		Consistently higher by ∼10%
	Yolk size	14.7% of the egg weight, or 17.5 g	12.3% of the egg weight, or 14.7 g
	Yolk Calcium and Phosphorus reserves	Higher Ca content, 29.28 ± 2.54 mg; Higher P content 106.73 ± 7.64 mg	Lower Ca content, 21.01 ± 1.65 mg; Lower P content, 81.35 ± 4.74 mg
Developmental dynamics	Eggshell thickness	Generally lower, no temporal trend observed	Decrease in thickness from d 11 to d 17
	Embryo weight	Progressively larger starting ED10	
	Ossification onset	ED11, no difference observed	
	Tibiotarsus	Longer and more robust before CAM* is established	No significant difference in length and volume after CAM is established
	Mandible	Slightly larger	
	Entire embryo	More advanced before CAM is established	Catches up in size and orientation after CAM is established

⁎ CAM: chorioallantoic membrane.

Our results (Table 1, Figure 1) confirm that the selection of breeders based on performance traits—such as meat production/muscle growth for Ross 308 chickens (Zuidhof, Schneider et al., 2014) and egg production/egg quality for Novoponte hens (Buzala and Janicki, 2016)—has been associated with differences in egg composition and embryo traits. The higher robustness of the eggshell in Novoponte eggs is consistent with selection for eggshell quality for table eggs (Figure 1D, Table 1). The higher yolk-to-white proportion of Ross 308 eggs correlates with the need for higher energy content (lipids) in Ross 308 eggs to match the metabolic expenditure of meat-type embryos (Sato, Tachibana et al., 2006, Druyan, 2010, Ho, Reed et al., 2011) (Table 1). The lower yolk weight in the layer chicken eggs is also consistent with the faster replacement egg yolk assembly observed in guillemots (Birkhead and del Nevo, 1987)—indeed, all eggs but first one produced by a layer hen are “replacement eggs”. Consequently, Ross 308 embryos are significantly larger than Novoponte embryos (Figure 1), which corroborates previous studies (Buzala and Janicki, 2016). The staining of embryos with Alcian Blue and Alizarin Red did not show significant differences in the onset of mineralization (Figure 2, red arrows pointing at pink, Alizarin Red positive diaphyseal segments in both strains, ED11), indicating that the skeleton ossification sequence and timing are conserved in the 2 strains.

### Two Sources of Calcium

Although layer hens produce substantially more eggs per unit of time, they still consistently assemble more robust mineralized eggshells, perhaps reflecting the selective breeding pressure, or a better-balanced specialized diet. Conversely, the thinner eggshell in broilers might be attributable to the leaner calcium reserves in the egg-laying female's medullary bone (Bloom, Domm et al., 1958): it is plausible that as a broiler hen's skeleton frame must support a heavier body, her dietary calcium is diverted away from medullary bone towards the rest of the skeleton, which is less specialized to function as a calcium depot for the eggshell mineralization. Since layer hens are selected for a higher laying rate and, therefore, a speedy assembly of the yolk (possibly, consistent with the observation in guillemots [Birkhead and del Nevo, 1987]), layer egg yolks are smaller than broiler egg yolks, the latter being assembled at a more physiologic rate.

To summarize, broiler embryos have a higher availability of yolk nutrients, including phosphorus and calcium, but lower eggshell mineral compared with layer embryos. Chicken embryos start to assimilate yolk nutrients, including mineral ions, during the first half of incubation, and proceed more rapidly during the second half of incubation (Noble and Cocchi, 1990). During the second half of incubation, the eggshell supplies calcium to the growing embryo via the chorioallantoic membrane (Gabrielli and Accili, 2010), which is fully functional and differentiated from day ED13 of incubation onwards (Makanya, Dimova et al., 2016). Such a switch in calcium procurement initially from the yolk in the first half of incubation, and then from the eggshell in the second half of the incubation period, may partly explain why the extent of mineralization of Novoponte embryonic skeletons appears to lag behind the mineralization of the Ross 308 embryonic skeletons on days ED11 and ED13. Conversely, the switch from the calcium source of the yolk to that of the shell around day ED13 can provide a plausible explanation why Novoponte embryonic skeletal development catches up (starting at day ED15) with their Ross 308 counterparts. Once the chorioallantoic membrane (**CAM**) becomes functionally active to lower local pH to dissolve calcium from the inner eggshell (thus decreasing the eggshell thickness over incubation time, Figure 3), the restriction in calcium resources is lifted for the Novoponte embryos, and ultimately a similar level of skeletal mineralization is reached for the 2 strains. This putative mechanism merits further investigation.

### Tibiotarsus as a Landmark of Skeletal Development

The tibiotarsus and the mandible are 2 of the first bones visibly ossified on day ED11, although cartilaginous anlagen are present earlier (Romanoff, 1960). The hindlimb develops ahead of the forelimb, which is attributable to evolutionary heritage from the clade of cursorial reptiles Archosauria—the common ancestor of birds and crocodiles (Bellairs and Jenkin, 1960). The tibiotarsus – like the majority of postcranial bones—develops through perichondral and endochondral ossification, beginning at the midshaft and progressing both towards the distal and proximal ends, which remain cartilaginous at the time of hatching. The mandible consists of 5 membranous bones: the dentary, splenial, angular, surangular, and prearticular, which eventually fuse at sutures. The 2 halves of the dentary bone fuse in the midline at the symphysis (Bellairs and Jenkin, 1960). The leveling of the tibiotarsus growth and mineralization pace differences between the 2 strains studied here supports the notion of highly conserved embryonic growth regulation.

### Supply and Demand Systems

The chicken is a precocial species, able to feed immediately after hatching. *Gallus gallus domesticus* chicks, as do other precocial species, demonstrate slower prehatching development of “supply” organs such as the gut, and rapid development of “demand” organs such as the brain, eyes and skeleton (Blom and Lilja, 2005). The equalization of tibiotarsus length and volume between layer and broiler, as observed here, might be linked to higher prioritization of the demand system—the skeleton—because precocity is attributable mainly to enhanced hindlimb growth (Atalgin and Kurtul, 2009). This finding is in accordance with prior reports indicating that the growth of bones with correct morphology is less contingent on the mineral ion supply than the extent of bone mineralization is (Williams, Solomon et al., 2000). The differences in the tibiotarsus between the 2 strains we examined are initially significant, but nearly vanish by day ED17, whereas the mandible is slightly smaller in the layer embryo by day ED17 (at least in our limited sampling of embryos). Perhaps the mandible difference may also vanish by the time of hatching, but that remains to be clarified.

## CONCLUSION

Eons of evolutionary refinement result in admirable robustness of biological regulation of growth and development in all organisms, birds included. Our results reveal that Ross 308 broiler eggs have a higher content of yolk nutrients including phosphorus and calcium, but lower eggshell mineral, compared to Novoponte layer eggs. By ED12, broiler embryos are significantly heavier than layer embryos. The higher content of yolk mineral ions (higher yolk-to-white ratio) in broiler eggs compared to layer eggs may partly explain the “head start” of skeletal mineralization that occurs in the broiler embryos (*e.g*., larger mandibles and tibiotarsi). However, the subsequent equalization of the skeletal mineralization in both strains starting by ED15, together with a steady decrease in the Novoponte eggshell thickness from ED11 to ED17, suggest a well-timed orchestrating role for the chorioallantoic membrane in directing mineral ion traffic from the dissolving shell to meet the high calcium demand required for skeletal mineralization as internal calcium becomes depleted from the yolk. Comparing the CAM activity in 2 strains would be of interest for future research. While there are minor timing differences in the skeletal developmental dynamics between broiler and layer embryos, we found no evidence that the selection of antagonist production traits affects the ultimate general robustness of prehatch skeletal mineralization. Since the extent of skeletal maturity equalizes between strains towards the end of incubation as governed by intrinsic robust biological regulation, refinements of farming practices, postnatal environment, and diet should be considered for improving domestic fowl welfare.

## DISCLOSURES

The authors declare that they have no known competing financial interests or personal relationships that could have appeared to influence the work reported in this paper.

## Appendix Supplementary materials

Image, video 1

Image, video 2

## Footnotes

Supplementary material associated with this article can be found in the online version at doi:10.1016/j.psj.2024.104308.
